# Central venous port-related infection in patients with malignant tumors: An observational study

**DOI:** 10.3109/03009734.2012.664178

**Published:** 2012-08

**Authors:** Akio Akahane, Miyuki Sone, Shigeru Ehara, Kenichi Kato, Michiko Suzuki, Ryoichi Tanaka, Akira Suwabe, Tetsuya Itabashi, Kashiwaba Masahiro

**Affiliations:** Department of Radiology, Iwate Medical University School of Medicine, 19-1 Uchimaru, Morioka 020-8505, Japan

**Keywords:** Central venous catheter, infection, venous port system

## Abstract

**Purpose:**

We evaluated the characteristics of central venous port (CVP)-related infection with microbiological assessments in patients with malignant tumors.

**Materials and methods:**

In a prospective setting, patients with CVP for the treatment of malignant tumors were enrolled in this study. The incidence of CVP-related infection during three months was determined. Microbiological surveillance from skin swab was performed before and after CVP placement.

**Results:**

Fifty-nine patients were enrolled in this study, and 60 CVPs were implanted. Thirty-six (61%) patients had head and neck malignancies. Access route was subclavian vein in 43 (71.7%) CVPs and forearm vein in 17 (28.3%). CVP-related infection was observed in three (5.1%) patients: port-pocket infection in one and probable CVP-related infection in two patients, respectively. No definitive CVP-related bloodstream infection was observed. Before the placement of CVP, colonization at the insertion site was observed in ten subclavian CVP patients, while no colonization was observed in the forearm CVP patients. At 1 and 4 weeks, detection rates of colonization were also higher in subclavian CVP patients. No definitive relationship was demonstrated between skin colonization and clinical development of CVP-related infection.

**Conclusion:**

The rate of CVP-related infection in this prospective evaluation in patients with malignant tumors was comparable to previous studies. Colonization of the skin was more prominent in the subclavian site than in the forearm site. Although skin colonization was not proven to be a risk factor of infection, these results may draw attention to the adequate maintenance of CVP. (Trial registration: UMIN000003664).

## Introduction

Central venous catheter (CVC)-related infection has been a frequent cause of hospital-acquired infection in patients with malignant tumors (1). Infection may lead to treatment delay and increase in patient morbidity and mortality. The reported incidence of CVC-related infections in patients with malignant tumor ranges from 7% to 19% (1,2).

The central venous port (CVP), a completely implantable device that enables repeated and long-term central venous access, was developed in the 1980s. During the past decade, the CVP has grown in importance in clinical practice in oncology for both emerging anticancer treatments and advances in supportive care. The CVP may have an advantage over the CVC in reducing the likelihood of contamination of the device by extraneous pathogens. However, infection remains a major problem in cancer patients who have undergone CVP implantation. The incidence of CVP-related infection varies between 0.9 and 10.1% (3–10). Most of the studies were retrospective investigations, and limited information regarding infection is available from prospective studies (5,7). Moreover, the indigenous bacterial flora of the overlying skin of the port may be associated with infectious adverse events (AEs); however, no data are available for this topic. The aim of this study was to evaluate the characteristics of CVP-related infections with microbiological assessments in patients with malignant tumors.

## Materials and methods

### Study design and patients

This study was a single-institute, prospective observational study on infectious AEs in patients with a central venous port system (CVP). The Institutional Review Board approved the study protocol before the initiation of patient enrollment. Between January 2010 and July 2010, eligible patients were included in this study and were prospectively observed for the study outcomes. Criteria for patient eligibility were as follows: hospitalized patient; 18 years old or older; indication for implantation of CVP for the treatment of a malignant tumor; follow-up was available at our institution; and written informed consent was obtained. Exclusion criteria included the following: pre-existing CVP, active inflammatory disease, and uncontrollable co-morbid diseases. All patients were informed of the complications and benefits of both chest port and arm port, and selected to the implantation site.

### Placement procedure of CVP

All CVPs were implanted via forearm or subclavian veins in the angiography suite by interventional radiologists or in the operating room by surgeons. Prophylactic antibiotics were not used. Maximal sterile barrier precautions using sterile gloves, gown, cap, mask, and a large drape were obtained throughout the procedure. We used 10% povidone-iodine for the sterilization. All venipunctures were made with an 18-G indwelling needle after subcutaneous administration of local anesthetic.

For the forearm approach, either the ulnar or radial antecubital vein was punctured. The ulnar vein was used when possible; however, the radial vein was accessed when the ulnar vein was narrow upon visual examination. Venography was not performed when selecting the vein to access. The forearm CVP was to be inserted on the opposite side of the dominant arm except in patients with only small-caliber veins on this side. A 5-Fr heparin-coated open-ended polyurethane catheter (Anthron PU; Toray Medical, Tokyo, Japan) was inserted over the guide wire, and the tip was placed at the level of junction of the superior vena cava and right atrium. After subcutaneous administration of local anesthetic, a pocket was created by making a 2–3-cm incision 1–3 cm peripheral to the venipuncture site.

For the subclavian approach, a 5-Fr heparin-coated polyurethane catheter (same catheter used in the forearm approach) or an 8-Fr valved silicone catheter (Groshong catheter; Bard Access Systems, Salt Lake City, UT, USA) was inserted over the guide wire, and the tip was placed at the level of the junction of the superior vena cava and right atrium. A pocket was created by making a 4–5-cm incision in the ipsilateral chest wall approximately 2 cm from the puncture site.

Implantation procedures, access routes, guiding method, and required time were recorded using dedicated case report forms.

### Maintenance of CVP

We did not have uniform hospital guidelines for the maintenance of CVP at the time of this investigation. In general, a port was punctured with a non-coring needle following sterilization with 10% povidone-iodine. A semipermeable transparent dressing was used to cover the needle and was fixed with adhesive tape. Needle insertion was performed when intravenous drip infusion was required. In patients with continuous or multiple infusion, a needle and an infusion line were exchanged every week. A total of 10 mL of 10% heparinized saline (100 IU/mL) was administered to lock the system before removal of the needle.

### Microbiological surveillance

We undertook microbiological surveillance at three time points: on the day of the placement of the CVP, 5–7 days after the placement, and 4 weeks after the placement. On the day of the placement of the CVP, two samples were obtained with a skin swab from an area about 4 cm in diameter at the insertion site of CVP before sterilization, and just after the CVP placement in a sterilized condition. At 5–7 days and 4 weeks after the placement, skin swabs were taken from the same area without sterilization. Micro-organisms were identified with Gram-stained smear examination. After searching 1000 fields per smear, samples were categorized using a five-grade scale: 0, ±, 1+, 2+, and 3+. We performed a qualitative analysis as follows: negative colonization, 0 or ±; positive colonization, 1+, 2+, or 3+. During episodes of fever (body temperature > 38.5°C) without any contributing sources other than the CVP, blood cultures were drawn from at least two sites: one via the CVP and the other by standard venipuncture. For cases of infectious signs around the port pocket, swab cultures were taken from either overlying skin or inside the pocket when removal was performed. Catheter-tip cultures were performed in patients with removal of CVP for the suspicion of CVP-related infections.

### Study outcomes

The main outcome measure was the incidence of CVP-related infection at 3 months after implantation of the system. According to the Centers for Disease Control and Prevention (CDC) guidelines for the prevention of CVC-related infections (11) and previous studies (12–14) regarding CVP-related infections, CVP-related infection was classified into three categories: CVP-related bloodstream infection, port-pocket infection, and probable CVP-related infection (Table I). The follow-up period was set at 3 months because the reported median time-to-infection ranged from 27 to 119 days in previous studies (4,15,16). The presence of the symptoms and signs of infection were routinely checked daily by the nursing staff, attending physicians, or the authors until discharge. Discharged patients were seen weekly or biweekly by attending physicians or the authors. The presence or absence of infection was recorded on a case report form of this study.

**Table I.** Definitions of the different types of CVP-related infection.

Definition	
CVP-related bloodstream infection
Defined as a combination of
1) Clinical features of infection, fever, and chills
2) Isolation of the same organism from the catheter tip and peripheral blood cultures
3) No other infectious focus explaining the positive blood culture result
Port-pocket infection	
1) Purulent discharge from the port pocket or other suspicious symptoms such as erythema, induration, or pain in the region of the port pocket
2) Isolation of the same organism from the catheter tip and from pus, with or without positive peripheral blood cultures
Probable CVP-related infection	
Defined as a combination of
1) Clinical features of infection, fever, and chills
2) Resolution of clinical sepsis after catheter removal
3) Absence of any other infectious focus
One of the following criteria was included:
a) Isolation of the organism from the peripheral blood cultures, but the catheter-tip culture was negative
b) Isolation of the same organism from peripheral blood cultures at a different time when fever and chills followed port flush, but the catheter-tip culture was negative
c) Blood cultures were negative or not performed

CVP = central venous port.

Secondary outcomes included technical success of CVP placement, types, and rates of non-infectious AEs, presence or absence of colonization and types of micro-organisms from skin swab cultures, and rates and reasons of CVP removal. Non-infectious AEs were categorized according to the Common Terminology Criteria for Adverse Events (CTCAE) v4.0 and reported if grade 2 or greater AEs were encountered. In patients with grade 2 or greater AEs before CVP placement, relevant AEs were recorded when the worsening of the grade was observed.

### Statistical considerations

Demographic and baseline variables were summarized by descriptive statistics. Incidences for each category of CVP-related infection were calculated as the number of first events over the number of patients at baseline. SPSS software, version 17 (SPSS, Chicago, IL, USA) was used for all analyses.

The sample size was considered ‘more is better’ regarding the nature of the observational study of frequency; however, we calculated the minimum required sample size in view of the feasibility of the study. We anticipated a 2.4% rate of CVP-related infection based on the median incidence from the literature. Calculating with a confidence interval of 95% and an interval estimation of 0.10, the minimum required sample was determined to be 55 (17). Thus, we set a sample size of 60 considering dropouts from the follow-up.

## Results

### Patient and treatment characteristics

Fifty-nine patients were enrolled in this study, and 60 CVPs were implanted: 58 patients had one CVP placement and 1 patient had two. Patient and treatment characteristics are reported in Tables II and III, respectively. Approximately two-thirds of the study population had head and neck malignancies. Forty-three (72.9%) patients underwent either chemotherapy or chemoradiotherapy as anticancer treatments. In all patients, planned follow-up was completed, and the intention-to-treat analysis was performed.

**Table II.** Baseline characteristics of patients (*n* = 59).

Characteristic	No. of patients (%)
Age, years	
Median	62
Range	24–85
Gender	
Male	41 (69.5)
Female	18 (30.5)
Primary tumor site	
Head and neck	36 (61.0)
Hematological	15 (25.4)
Gastrointestinal	4 (6.8)
Breast	3 (5.1)
Lung	1 (1.7)
Host risk factor	
Diabetes	3 (5.1)
Leukopenia^a^	5 (8.5)
Therapeutic risk factor	
Urinary catheter	0 (0)
Tracheostomy	9 (15.3)
Chest drainage tube	1 (1.7)
Prior antibiotics	10 (16.9)
Steroid use	4 (6.8)
Mean laboratory values	
Albumin, g/dL	3.5
Hemoglobin, g/dL	8.3

^a^WBC < 3000 μL.

**Table III.** Treatment characteristics.

Treatment	No. of patients (%)
Chemoradiotherapy	32 (54.2)
Chemotherapy alone	11 (18.6)
Radiotherapy alone	0 (0)
Stem cell transplantation	1 (1.7)
Surgery	20 (33.9)
Palliative treatments	8 (13.6)

### Implantation of CVP

Technical success of CVP placement was achieved in all patients (100%). Details of the placement procedure are shown in Table IV. The access route was the subclavian vein in 43 (71.7%) CVPs, and 36 (60%) CVPs were placed by interventional radiologists in the angiography suite. All forearm CVP placements were performed by interventional radiologists. Periprocedural AEs are addressed in the following section of non-infectious AEs.

**Table IV.** Details of CVP placement.

Parameter	No. of patients (%)
Insertion site	
Subclavian	43 (71.7)
Forearm	17 (28.3)
Side	
Right	38 (63.3)
Left	22 (36.7)
Procedure place	
Operating room	7 (11.7)
Angiography suite	53 (88.3)
Guiding method	
Ultrasound and fluoroscopy	36 (60.0)
External landmark	24 (40.0)
Type of catheter	
Open-ended	57 (95.0)
Valved	3 (5.0)
Procedure time, minutes	
10–30	36 (60.0)
31–45	16 (26.7)
46–60	8 (13.3)
Operator experience, cases	
<10	11 (18.3)
10–50	10 (16.7)
51–100	28 (46.7)
101–200	9 (15.0)
>200	2 (3.3)

The denominator for the percentage is the total number of procedures (*n* = 60).

The cumulative port access period was 2038 days (range 0–90 days, median 32.5 days), and port puncture occurred 263 times (range 0–20, median 4). In three patients, CVP was not used during the study period due to the alteration of the treatment from systemic chemotherapy to oral chemotherapy or the extension of parenteral nutrition.

### CVP-related infection and colonization

CVP-related infection was observed in three patients (5.1%): port-pocket infection was observed in one patient (1.7%), and probable CVP-related infection was found in two patients (3.4%). Summaries of the characteristics of patients and infection are listed in Tables V and VI, respectively. All patients had head and neck malignancies. In two patients, definitive CVP-related culture findings were not observed; however, fever and chills were observed following the flush of a port and categorized as probable CVP-related infection. The port access periods of the patients were 60, 80, and 45 days, and the number of port punctures were 8, 10, and 5 times, respectively. These three patients were successfully treated with the removal of CVP and the administration of antibiotics. No definitive CVP-related bloodstream infection was observed in this study.

**Table V.** Summary of CVP-related infection: characteristics of patients (*n* = 3).

					Laboratory data (lowest values)						
	Age	Sex	Primary tumor site	Risk factor	Albumin, g/dL	Hemoglobin, g/dL	Lymphocyte/μL	Insertion site	Treatment characteristics	Insertion site	Type of catheter
1	74	M	Gingiva	Tracheostomy	1.9	7.3	350	Subclavian	Chemoradiotherapy	Subclavian	Open-ended
2	70	M	Mandible	None	2.5	8.1	220	Subclavian	Chemoradiotherapy	Subclavian	Open-ended
3	62	M	Larynx	Steroids, prior antibiotics	2.7	10.1	360	Forearm	Chemoradiotherapy	Forearm	Open-ended

**Table VI.** Summary of CVP-related infection: characteristics of infection (*n* = 3).

			Micro-organism in the insertion site on surveillance			Micro-organisms at removal of CVP		
Patient	Type	Onset, days	Pre-CVP placement	1 week	4 weeks	Port pocket	Catheter tip	Peripheral blood
1	Port-pocket infection	66	Negative	Negative	*Corynebacterium*	*S. aureus*	*S. aureus*	Negative
2	Probable CVP-related infection	94	CNS, *Corynebacterium*	*Corynebacterium*	Negative	Negative	Negative	Negative
3	Probable CVP-related infection	69	Negative	Negative	Negative	Negative	Negative	MRSA

CNS = coagulase-negative staphylococci; *S. aureus* = *Staphylococcus aureus*; MRSA = methicillin-resistant *Staphylococcus aureus*.

Colonization at the insertion site of CVP was observed only in ten subclavian port patients (Table VII) before sterilization on the day of CVP placement. After sterilization, only one patient showed colonization (Table VII). At baseline, indigenous skin bacteria (i.e. *Staphylococcus aureus* and coagulase-negative staphylococci) were the frequently detected micro-organisms (18.6%). At 1 and 4 weeks after placement, detection rates of all micro-organisms were also higher in subclavian CVP patients than in forearm CVP patients. Colonization at 1 and 4 weeks was observed in 10 head and neck cancer patients out of a total of 11 patients.

**Table VII.** Colonization at the insertion site of subclavian and forearm CVPs (*n* = 59).

	Subclavian: No. of patients (%)				Forearm: No. of patients (%)			
	On the day of placement		1 week	4 weeks	On the day of placement		1 week	4 weeks
	Pre-sterilization	Post-sterilization			Pre-sterilization	Post-sterilization		
Gram-positive cocci	10 (23.3)	1 (2.3)	8 (18.6)	8 (18.6)	0	0	1 (5.9)	2 (11.8)
*Staphylococcus aureus*	0	0	1 (2.3)	0	0	0	1 (5.9)	0
CNS	8 (18.6)	1 (2.3)	5	6	0	0	0	2 (11.8)
Enterococci	0	0	0	0	0	0	0	0
Other cocci	2 (4.7)	0	2 (4.7)	2 (4.7)	0	0	0	0
Gram-negative bacilli	0	0	0	1 (2.3)	0	0	0	0
Yeasts	0	0	0	0	0	0	0	0
Others	0	0	0	0	0	0	0	0
Total	10 (23.3)	1 (2.3)	8 (18.6)	9 (20.9)	0	0	1 (5.9)	2 (11.8)

CNS = coagulase-negative staphylococci.

### Non-infectious AEs

Table VIII lists non-infectious AEs that occurred during and after CVP placement. During the placement procedure, we did not encounter severe AEs. Three patients developed hematoma at the port pocket, which was treated with compression and needle drainage but did not require continuous drainage or further hemostatic treatments. Five patients experienced non-infectious postprocedural AEs. Phlebitis and system occlusion were observed in forearm port patients, and venous thrombosis and pulmonary thromboembolism were observed in a subclavian port patient. All instances of phlebitis were recorded within 1 week after insertion. In patients with system occlusion, recanalization was successfully performed by injecting a mixture of 60,000 IU urokinase and 5,000 IU heparin through a port. In one patient with venous thrombosis and pulmonary thromboembolism, we removed the CVP immediately after the diagnosis and treated the patient with anticoagulants. We did not observe any CVP-related death in this study.

**Table VIII.** Non-infectious AEs.

AE	No. (%)
Periprocedural	
Pneumothorax	0 (0)
Arterial puncture	0 (0)
Hematoma	3 (5.1)
Total	3 (5.1)
Postprocedural	
Phlebitis	3 (5.1)
Fibrin sheath	0 (0)
System occlusion	1 (1.7)
Subcutaneous extravasation	0 (0)
Venous thrombosis	1 (1.7)
Pulmonary thromboembolism	1 (1.7)
Catheter detachment	0 (0)
Total	6 (10.1)

### Removal of CVP

We removed the CVP in seven patients (11.7%). Emergency removal for AE was needed in five patients (8.3%). In the emergency patients, the median time-to-removal was 65 days (range 34–94 days) (Table IX).

**Table IX.** Removal of CVP.

Parameter	No. of patients (%)
Removal of CVP	7 (11.7)
Indication for removal:	
Infection (total)	3 (5.1)
CVP-related bloodstream infection	0 (0)
Port-pocket infection	1 (1.7)
Probable CVP-related infection	2 (3.4)
Catheter occlusion	0 (0)
Pulmonary thromboembolism	1 (1.7)
Wound disruption	1 (1.7)
No longer needed	2 (3.4)
Median dwell time, days (range)	66 (34–103)

The denominator for the percentage is the total number of patients (*n* = 59).

## Discussion

The incidence of CVP-related infection in the literature varies between 0.9 and 10.1% (3–10), and most of the studies were retrospective investigations. In this prospective study, 3 out of 59 patients (5%) presented CVP-related infection. At the surveillance of the overlying skin of the port, the subclavian site was associated with a higher incidence of colonization than the forearm site, both before implantation (23.3% versus 0%) and at 1 week (18.6% versus 5.9%) and 4 weeks after implantation (20.9% versus 11.8%). However, no definitive relation was observed between the presence of colonization and the development of infection.

According to the guidelines from the CDC (11), the density of skin flora at the catheter insertion site is a major risk factor for catheter-related bloodstream infections of CVC. The subclavian site is preferred instead of a jugular or femoral site to reduce the risk for infection because of a lower density of skin flora (18–20). In the setting of CVP, skin flora may also contribute to infection because repeated puncture is performed through the overlying skin of the port, although no data are available from the literature. In our study, more colonization was observed at the subclavian site than at the forearm site. Sadoyama et al. demonstrated that more colonization was observed at the subclavian site than at the jugular site in patients with CVCs at the intensive care unit (21). The subclavian site may be more vulnerable to skin flora than previously recognized; however, no definitive relevance with clinical infection was demonstrated in our study.

The incidence of CVP-related infection of 5% in our study is consistent with that reported in other studies (3,7,22–24). In our study, all cases of infection were observed in head and neck cancer patients. We could not eliminate selection bias. Because of the referral pattern, two-thirds of our cohort constituted head and neck cancer patients. Previous studies revealed an infection rate of 8.0%–8.4% in head and neck cancer patients (25,26), and this population may be at risk of CVP-related infection. Hematologic malignancies may also be a risk factor for infection because of intensive chemotherapies resulting in neutropenia. In our study, however, no infection was observed in patients with hematologic malignancy. Moreover, regarding the incidence of CVP-related infection, adequate diagnosis and classification are important because the reported diagnostic criteria of infection varied among studies (3,5,24) and may result in uncertainty in comparison. In our study, evaluation of infection was performed with rigorous methods to obtain reliable results.

Various factors may contribute to the development of infection. Possible factors include the general status of the patients, therapeutic regimen, materials making up the catheter, placement procedures, and maintenance procedures. Maintenance of CVPs differs from that of other long-term venous devices such as CVCs. A reservoir in a port enables the CVP to be completely implanted under the skin and may reduce the risk of infection. However, multiple and somewhat complicated maintenance procedures are required for CVPs, which may increase the risk of contamination. Factors associated with the risk of infection at maintenance include the timing and duration of needle insertion, aseptic techniques, dressing, management of lines and hubs, and the use of prophylactics for venous thrombosis. Although definitive evidence is not established for each of these factors, sensible measures against these issues are mandatory. We have been developing a uniform protocol for the maintenance of CVP in our hospital. In particular, review and revision of the management of subclavian CVP are considered important based on the microbiological results of this study.

According to a review by Vescia et al. (27), removal of the CVP is not routinely recommended in patients with CVP-related infections. The CVP must be removed for patients with instability, systemic complications from infection, signs of port-pocket infection, persistent sepsis or relapse of infection after antibiotic treatment, or the detection of certain micro-organisms resistant to antimicrobial treatments with catheter salvage (e.g. *S. aureus* or *Candida* species). In the guidelines for CVCs, prophylactic antimicrobial therapy is not recommended (11). The efficacy of antibiotic lock of the CVP with a high-dose solution of antibiotics for treatment and prevention of infection remains controversial and is not routinely recommended (28–30). In our study, all three patients underwent catheter removal (port-pocket infection in one patient and unstable patient condition in two patients), and the patients recovered after the removal.

Several limitations of our study warrant comments. First, the cohort size was small, and the observational period was not long. The number of patients with infection of three is not sufficient to perform statistical analyses for risk factors of infection. Second, the patients were not adjusted regarding the tumor type or other factors because of the limitation of a single-arm observational study. Third, uninvestigated confounding factors may contribute to infection. Maintenance of CVP during the follow-up period certainly is the main uninvestigated confounding factor in this study. Optimization of the maintenance protocol is needed in future studies.

In conclusion, the rate of CVP-related infection in this prospective evaluation in patients with malignant tumors was comparable to that reported in previous studies. Colonization of the skin was more prominent in the subclavian site than in the forearm site. Although skin colonization was not proven to be a risk factor for infection, these findings serve to draw attention to the adequate maintenance of CVP.

## References

[1] Groeger JS, Lucas AB, Thaler HT, Friedlander-Klar H, Brown AE, Kiehn TE (1993). Infectious morbidity associated with long-term use of venous access devices in patients with cancer. Ann Intern Med.

[2] Carratala J, Niubo J, Fernandez-Sevilla A, Juve E, Castellsague X, Berlanga J (1999). Randomized, double-blind trial of an antibiotic-lock technique for prevention of gram-positive central venous catheter-related infection in neutropenic patients with cancer. Antimicrob Agents Chemother.

[3] Goltz JP, Scholl A, Ritter CO, Wittenberg G, Hahn D, Kickuth R (2010). Peripherally placed totally implantable venous-access port systems of the forearm: clinical experience in 763 consecutive patients. Cardiovasc Intervent Radiol.

[4] Inaba Y, Yamaura H, Sato Y, Najima M, Shimamoto H, Nishiofuku H (2007). Central venous access port-related complications in outpatient chemotherapy for colorectal cancer. Jpn J Clin Oncol.

[5] Biffi R, Pozzi S, Agazzi A, Pace U, Floridi A, Cenciarelli S (2004). Use of totally implantable central venous access ports for high-dose chemotherapy and peripheral blood stem cell transplantation: results of a monocentre series of 376 patients. Ann Oncol.

[6] Marcy PY, Magne N, Castadot P, Italiano A, Amoretti N, Bailet C (2007). Is radiologic placement of an arm port mandatory in oncology patients? Analysis of a large bi-institutional experience. Cancer.

[7] Vardy J, Engelhardt K, Cox K, Jacquet J, McDade A, Boyer M (2004). Long-term outcome of radiological-guided insertion of implanted central venous access port devices (CVAPD) for the delivery of chemotherapy in cancer patients: institutional experience and review of the literature. Br J Cancer.

[8] Bodner LJ, Nosher JL, Patel KM, Siegel RL, Biswal R, Gribbin CE (2000). Peripheral venous access ports: outcomes analysis in 109 patients. Cardiovasc Intervent Radiol.

[9] Lorch H, Zwaan M, Kagel C, Weiss HD (2001). Central venous access ports placed by interventional radiologists: experience with 125 consecutive patients. Cardiovasc Intervent Radiol.

[10] Kawamura J, Nagayama S, Nomura A, Itami A, Okabe H, Sato S (2008). Long-term outcomes of peripheral arm ports implanted in patients with colorectal cancer. Int J Clin Oncol.

[11] O'Grady NP, Alexander M, Dellinger EP, Gerberding JL, Heard SO, Maki DG (2002). Guidelines for the prevention of intravascular catheter-related infections. Infect Control Hosp Epidemiol.

[12] Liaw CC, Chen JS, Chang HK, Huang JS, Yang TS, Liau CT (2008). Symptoms and signs of port-related infections in oncology patients related to the offending pathogens. Int J Clin Pract.

[13] van Rooden CJ, Schippers EF, Guiot HF, Barge RM, Hovens MM, van der Meer FJ (2008). Prevention of coagulase-negative staphylococcal central venous catheter-related infection using urokinase rinses: a randomized double-blind controlled trial in patients with hematologic malignancies. J Clin Oncol.

[14] Cesaro S, Tridello G, Cavaliere M, Magagna L, Gavin P, Cusinato R (2009). Prospective, randomized trial of two different modalities of flushing central venous catheters in pediatric patients with cancer. J Clin Oncol.

[15] Funaki B, Szymski GX, Hackworth CA, Rosenblum JD, Burke R, Chang T (1997). Radiologic placement of subcutaneous infusion chest ports for long-term central venous access. AJR Am J Roentgenol.

[16] Beckers MM, Ruven HJ, Seldenrijk CA, Prins MH, Biesma DH (2010). Risk of thrombosis and infections of central venous catheters and totally implanted access ports in patients treated for cancer. Thromb Res.

[17] Machin D, Campbell MJ, Tan SB, Tan SH (2008). Sample size table for clinical studies.

[18] Mermel LA, McCormick RD, Springman SR, Maki DG (1991). The pathogenesis and epidemiology of catheter-related infection with pulmonary artery Swan-Ganz catheters: a prospective study utilizing molecular subtyping. Am J Med.

[19] Goetz AM, Wagener MM, Miller JM, Muder RR (1998). Risk of infection due to central venous catheters: effect of site of placement and catheter type. Infect Control Hosp Epidemiol.

[20] Richet H, Hubert B, Nitemberg G, Andremont A, Buu-Hoi A, Ourbak P (1990). Prospective multicenter study of vascular-catheter-related complications and risk factors for positive central-catheter cultures in intensive care unit patients. J Clin Microbiol.

[21] Sadoyama G, Gontijo Filho PP (2003). Comparison between the jugular and subclavian vein as insertion site for central venous catheters: microbiological aspects and risk factors for colonization and infection. Braz J Infect Dis.

[22] Kock HJ, Pietsch M, Krause U, Wilke H, Eigler FW (1998). Implantable vascular access systems: experience in 1500 patients with totally implanted central venous port systems. World J Surg.

[23] McNulty NJ, Perrich KD, Silas AM, Linville RM, Forauer AR (2009). Implantable subcutaneous venous access devices: is port fixation necessary? A review of 534 cases. Cardiovasc Intervent Radiol.

[24] Charvat J, Linke Z, Horaekova M, Prausova J (2006). Implantation of central venous ports with catheter insertion via the right internal jugular vein in oncology patients: single center experience. Support Care Cancer.

[25] Gruber B, Moran WJ, Vokes EE, Dobleman TJ, Shaw GY, Guarnieri CM (1988). Implantable venous access device: use in patients with advanced head and neck cancer. Otolaryngol Head Neck Surg.

[26] Akahane A, Sone M, Ehara S, Katoh K, Tanaka R, Nakasato R (2011). Subclavian vein versus arm vein for totally implantable central venous port for head and neck cancer patients: a retrospective comparative analysis. Cardiovasc Intervent Radiol.

[27] Vescia S, Baumgartner AK, Jacobs VR, Kiechle-Bahat M, Rody A, Loibl S (2008). Management of venous port systems in oncology: a review of current evidence. Ann Oncol.

[28] Rubie H, Juricic M, Claeyssens S, Krimou A, Lemozy J, Izard P (1995). Morbidity using subcutaneous ports and efficacy of vancomycin flushing in cancer. Arch Dis Child.

[29] Del Pozo JL, Garcia Cenoz M, Hernaez S, Martinez A, Serrera A, Aguinaga A (2009). Effectiveness of teicoplanin versus vancomycin lock therapy in the treatment of port-related coagulase-negative staphylococci bacteraemia: a prospective case-series analysis. Int J Antimicrob Agents.

[30] Safdar N, Maki DG (2006). Use of vancomycin-containing lock or flush solutions for prevention of bloodstream infection associated with central venous access devices: a meta-analysis of prospective, randomized trials. Clin Infect Dis.

